# ERP correlates of intramodal and crossmodal L2 acquisition

**DOI:** 10.1186/1471-2202-12-48

**Published:** 2011-05-25

**Authors:** Nils Skotara, Monique Kügow, Uta Salden, Barbara Hänel-Faulhaber, Brigitte Röder

**Affiliations:** 1 Biologische Psychologie & Neuropsychologie, Universität Hamburg, Von-Melle-Park 11, 20146 Hamburg, Germany; 2 Sonderforschungsbereich 538 Mehrsprachigkeit, Universität Hamburg, Max-Brauer-Allee 60, 22765 Hamburg, Germany; 3 Erziehungswissenschaften, Sektion II: Wahrnehmung & Kommunikation, Universität Hamburg, Sedanstr. 19, 20146 Hamburg, Germany

## Abstract

**Background:**

The present study compared the neural correlates of an intramodally and a crossmodally acquired second language (L2). Deaf people who had learned their L1, German Sign Language (DGS), and their L2, German, through the visual modality were compared with hearing L2 learners of German and German native speakers. Correct and incorrect German sentences were presented word by word on a computer screen while the electroencephalogram was recorded. At the end of each sentence, the participants judged whether or not the sentence was correct. Two types of violations were realized: Either a semantically implausible noun or a violation of subject-verb number agreement was embedded at a sentence medial position.

**Results:**

Semantic errors elicited an N400, followed by a late positivity in all groups. In native speakers of German, verb-agreement violations were followed by a left lateralized negativity, which has been associated with an automatic parsing process. We observed a syntax related negativity in both high performing hearing and deaf L2 learners as well. Finally, this negativity was followed by a posteriorly distributed positivity in all three groups.

**Conclusions:**

Although deaf learners have learned German as an L2 mainly via the visual modality they seem to engage comparable processing mechanisms as hearing L2 learners. Thus, the data underscore the modality transcendence of language.

## Background

Language acquisition has often been studied by comparing native speakers with people who have learned a second language (L2) later in life. Central to many such studies is the hypothesis that sensitive or even critical periods exist in neural system development that provide windows of greater potential for learning. Sensitive periods create particularly appropriate conditions for the acquisition of new skills and abilities. Later in life, for many functional systems, this early plasticity is reduced or absent [e.g. [1]]. In line with this hypothesis, many studies investigating the language abilities of L2 learners who learned a language late - for example after 16 years of age [2] - have repeatedly documented poorer performances and different neural correlates of language processing compared to native speakers of the same language. Further, behavioural studies have shown that syntax and phonology pose greater challenges for L2 learners than lexical-semantic aspects [3].

As many L2 speakers have learned the language after an assumed sensitive/critical period, factors related to Age of Acquisition (AoA) have been found to explain the lower performance level to a large degree. Indeed Wartenburger et al. [4] reported that the neural correlates of syntactic processing predominantly depend on AoA. By contrast the neural correlates of semantic processing were mainly defined by the proficiency level of the L2 learners. Brain activations related to semantic processing as a function of proficiency were also found by Lee et al. [5] and Perani et al. [6] who postulated that the attained proficiency might better explain differences of the cortical representation among L2 learners than AoA.

Another, less often investigated, factor that might influence L2 acquisition is the modality through which the L1 and the L2 have predominantly been acquired. Congenitally deaf humans born to deaf parents learn both their L1 and L2 exclusively through the visual modality. Thus, such individuals provide a unique opportunity to investigate the influence of the L1 modality on proficiency and neural correlates of the L2. This approach allows for investigating modality invariant aspects of human language.

In the ongoing debate on language acquisition of deaf people, some authors have argued that sign languages and spoken languages compete for resources associated with language processing on a neuronal level [5]. Therefore, the early acquisition of a signed L1 during the sensitive/critical period may occupy crucial brain systems necessary for learning a written language. Advocates of this hypothesis have argued that language acquisition strongly depends on the modality through which the language has been mediated. In line with this perspective, it is possible to assume that the acquisition of a sign language prevents a successful subsequent acquisition of a spoken language later in life. Therefore, advocates of the modality competition hypothesis would suggest that deaf children should not learn a sign language first, but rather acquire the language being spoken in their environment in the first place. However, the neural processing mechanisms of L2 speakers as a function of the modality of the L1 are yet almost unknown. The finding of different processing mechanisms for the L2 in deaf L2- and hearing L2 users would argue in favour of the competition hypothesis, while the finding of comparable processing mechanisms for language comprehension in these two groups would argue against the modality competition hypothesis. Specifically, the competition hypothesis would predict the absence of typical language-related event related potentials (ERPs) in native signers when they process written German.

Deaf people are not able to learn a spoken L2 by hearing; they acquire German only through the visual modality. This leads to an acquisition of German without a representation of the typical sounds. We call the acquisition of German (as an oral language) by deaf L2 learners crossmodal acquisition, whereas the acquisition of German by hearing L2 learners is called intramodal. A comparable ERP pattern for crossmodal and intramodal L2 acquisition would strongly argue for a development of language related processes independent of its modality.

In summary, the present study compared written German language processing of hearing and deaf L2 learners of German. The groups were matched for AoA of their L2. ERPs of a group of hearing native speakers of German were used to assess general effects of L2 acquisition.

### Semantic and syntactic processing in native speakers

Neural correlates of language processing are often investigated using a violation paradigm. In this paradigm, two otherwise identical sentences differ in only one word, which is correct or either semantically or syntactically incorrect. Sentences of both types are presented via the visual or auditory modality while the electro-encephalogram (EEG) is simultaneously recorded. The difference in the average brain response to the incorrect and correct words is analyzed: Semantic processes have commonly been associated with the N400 effect, a negativity which has most often been observed with a centroparietal maximum for written languages [7]. The amplitude of the N400 effect is inversely correlated with lexical expectancy and is considered to reflect lexical semantic integration processes [8-10].

A series of recent studies have reported that semantic violations within the arguments of the verb sometimes evoke positive waves following the N400 in a sentence that is syntactically well formed [9,11-13]. It has been suggested that an enhanced complexity of such sentences causes enhanced processing demands that might be reflected in these late positivities [14].

Syntactic processes have been associated with early and later ERP effects in written languages. The early effect, the left anterior negativity (LAN), has been found to start at around 300 ms following the presentation of different kinds of syntactic anomalies, such as morphological congruency violations [15-18]. It has been suggested that the LAN either reflects genuine syntactic parsing processes [19] and/or predominantly working memory functions related to complex processing operations [20]. It seems to indicate a rule-based mechanism related to a morphosyntactic anomaly detection [19,21] and has been found to vary with the severity of a particular mismatch [22,23].

Following this negativity, a posteriorly distributed positivity, which has been called the syntactic positive shift (SPS) [24] or (henceforth) P600 [25], has been observed after anomalies in the phrase-structure of the sentence, garden-path sentences and after morphosyntactic violations. This ERP has been considered to reflect the processing costs of the reanalysing process activated when an anomaly is detected [e.g. [23]] or genuine parsing processes.

Of special interest for the present study are ERP experiments manipulating subject-verb number agreement in written languages [18,22-24,26]. Kaan [26] investigated the ERP correlates of violations of subject-verb number agreement in Dutch. She found a LAN-like effect for the time period between 300 and 500 ms after the critical word. This negativity, however, had a bilateral rather than left lateralized scalp distribution with a central maximum. Hagoort et al. [24] reported a P600 following subject-verb agreement violations, but did not analyse the time period between 0 and 500 ms after stimulus onset. By contrast, Osterhout and Mobley [18], Münte et al. [23], and De Vicenzi et al. [22] reported both a LAN and P600 for subject-verb number agreement violations in English, German and Italian, respectively. Kaan's [26] finding of a bilaterally distributed LAN-like negativity is no exception. Numerous studies with native speakers reported a distribution of the LAN effect, which was neither strictly left nor anterior: Rodriguez-Fornells, Clahsen et al. [21], in Catalan, and Osterhout and Mobley [18], in English, found a left temporal topography for syntactic violations; Gross, Say et al. [27] observed a right anterior maximum for syntactic violations in Italian; Weyerts, Penke et al. [28], and Münte et al. [23] reported a bilateral frontocentral distribution for syntactic violations in German.

### ERP comparisons between L1 and L2 speakers

Many studies have consistently shown a number of differences when comparing ERPs to an L1 and an L2. These studies have demonstrated, in line with behavioural data, that semantic processing (N400) seems to be less vulnerable to a delayed language acquisition than syntactic processing (LAN and P600). Thus, the ERP patterns observed to an L2 differ more from those to a native language for syntactic processes than for semantic processes:

Weber-Fox and Neville [2] investigated Chinese L2 learners of English with different AoA. A native-like functional organization of the brain for syntactic processes was found only in L2 learners with an AoA below four years. Hahne and Friederici [29] investigated Japanese L2 learners of German using phrase structure violations. In the native speakers, an incorrect compared to a correct sentence elicited an early LAN (ELAN) between 100-250 ms and a P600 between 500-1000 ms, by contrast neither an ELAN nor a P600 were observed in the L2 group.

Chen et al. [30] demonstrated that Chinese L2 learners of English, who were able to correctly detect subject-verb agreement violations, displayed an ERP pattern different to that of native speakers of English. While they observed a LAN followed by a P600 for native speakers, they found a central anterior negativity between 500 and 700 ms followed by a P600 for Chinese L2 learners of English. The results suggest that L2 learners might use different processing strategies to achieve similar performance levels as native speakers. These differences between L1 and L2 learners associated with syntactic violations have been interpreted as the result of a reduced automaticity in L2 processing. This in turn may be due to an additional drain on working memory processing, since, in contrast to L1 learners, L2 learners must explicitly recapitulate the words and phrases in the L2 [31]. This hypothesis was supported by a functional Magnetic Resonance Imaging (fMRI) study that demonstrated a higher activity in a widespread cortical network shared by both languages during the processing of an L2 compared to an L1 [32]. Late L2 learners have been hypothesized to rely more on the declarative memory system as opposed to L1 speakers who have been assumed to rely on procedural memory processes [33] during language comprehension.

However, these interpretations have been questioned for a number of reasons as many studies have reported a variety of other influences that might contribute to differences between L1 and L2 speakers: In a study by Osterhout et al. [34], native speakers of French showed a P600 after subject-verb agreement violations in their native language, whereas for English L2 learners of French an N400-like effect was observed after the same violations. Interestingly, after eight months of training, the L2 learners displayed a P600 to syntactic phenomena which are similar in French and English, but not to syntactic phenomena which are specific to French. Such findings suggest that differences in L2 learning might depend on similarity between the L1 and L2 and proficiency level of the participants. Indeed, Tokowicz and MacWhinney [35], who investigated English (L1) speaking learners of Spanish (L2), demonstrated that a subject-verb agreement violation elicited a P600 both for English and Spanish. By contrast a determiner-noun agreement violation did not elicit a P600 effect in the L2 (see Hahne et al [36] for similar findings in Russian L2 learners). However, Clahsen and Felser [37] proposed that more subtle differences might emerge, regardless of the proficiency level if syntactic structures of higher complexity were used. Indeed, even highly proficient L2 learners of English, with different L1s, did not process so called non-local grammatical phenomena in a native-like manner (that is grammatical constructions which can only be judged by processing items across phrase boundaries). Further, a different involvement of executive processes [38], higher between participant variability [39], and different methods of training and allocation of attention [40] might contribute to differences in the neural correlates and proficiency of L1 and L2 learners.

### Sign language processing

As in any natural language, different national languages and even different dialects exist in sign languages [41]. Sign languages have evolved naturally in a similar way as spoken languages and continue to do so [41]. They exhibit the same linguistic structures as any natural language at all linguistic levels such as phonology, morphology, syntax and semantics [42,43]. Even the time course of sign language development in children appears to be very similar to spoken languages [44,45]. Numerous studies including fMRI [46,47], neuropsychological [48,49], and ERP studies [50,51] have provided evidence for similar processing systems in oral and sign language comprehension [52]. For example, fMRI studies have demonstrated similar activation patterns for both types of languages [46,47,53].

### Deaf people as L2 learners

Behavioural studies have shown that congenitally deaf people who had learned a signed language (American Sign Language, ASL) as an L1 on schedule perform better in English (L2) than congenitally deaf people who had learned ASL as an L1 later in life (they started between 6-13 years) [54,55]. Most importantly, the first group did not differ in proficiency from hearing people who had learned English as an L2 after the acquisition of an oral L1 (Boudreault and Mayberry 2006; Mayberry and Lock 2003). These results provide strong evidence for the assumption that the acquisition of an L1 during the sensitive/critical period is, regardless of its modality, a necessary foundation for learning other languages later in life. Similar as for hearing L2 learners, deaf L2 (English) learners were hardly distinguishable from native speakers in their ERP correlates of semantic processing [51,56].

### Predictions

The present study compared intramodal and crossmodal L2 acquisition. A group of hearing native speakers of German served as a control group (henceforth called h-L1) in order to compare them with hearing L2 learners of German (henceforth called h-L2) and congenitally deaf people who had started learning written German as an L2 (henceforth called d-L2) at a comparable age. In line with the recent literature [54], we expected d-L2 to perform at a comparable level as h-L2. Both L2 groups were expected to perform worse than h-L1, particularly in the syntactic condition.

For h-L1, we expected to observe a centroparietally distributed N400 effect after semantically implausible words. Following verb-agreement violations, we predicted a left anterior negativity (LAN) and a posteriorly distributed positivity (P600).

Since we expected syntactic processing to be more vulnerable to late acquisition than semantic processing, we anticipated a lack of or a different topography for the early syntactic negativity in h-L2. Since a P600 has often been found in hearing L2 learners as well [36], we anticipated h-L2 to show a P600 effect in the syntactic conditions. For the semantic condition, a similar N400 effect was predicted for h-L2 as for h-L1.

Similar ERP patterns for d-L2 and h-L2 would indicate similar brain mechanisms involved in intramodal and crossmodal L2 acquisition. By contrast, if a signed L1 modified the functional cerebral organization of language, we would expect the ERP patterns for h-L2 and d-L2 to differ significantly.

## Results

### Behavioural Data

**Figure 1:**
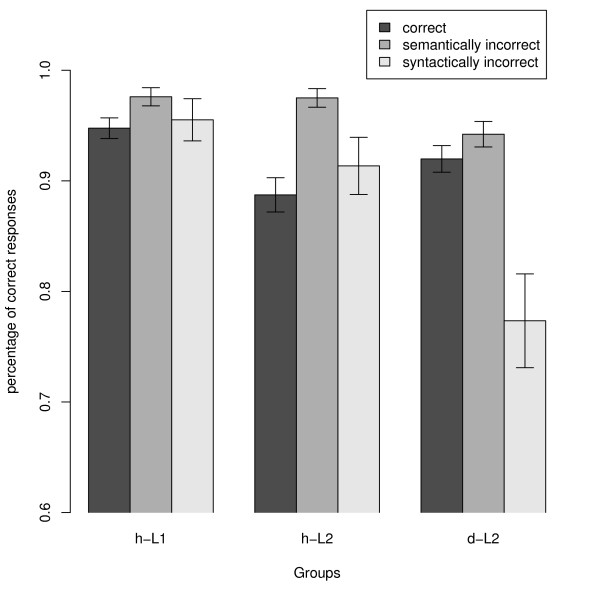
**Behavioural results**. Percentages of correct responses for each group for correct, semantically, and syntactically incorrect sentences. From left to right: hearing native speakers (h-L1), hearing L2 learners (h-L2), and deaf L2 learners (d-L2).

The mean percentages of correct responses are shown in figure 1 and table 1. The statistical analysis revealed a main effect of Group (F(2, 29) = 8.686; p < 0.001; mean percentages of correct responses: h-L1: 96.0% (SE: 0.8%), h-L2: 92.5% (SE: 1.5%), and d-L2: 87.8% (SE: 1.6%)). Bonferroni-corrected post-hoc tests revealed a significantly higher performance level for h-L1 than for d-L2 (t(9.967) = 4.514, p = 0.003). The remaining pair comparisons were not significant. Furthermore, a main effect of Condition (F(1.4, 39.6) = 16.536; ε = 0.684; p < 0.001) and an interaction of Group and Condition (F(2.7, 39.6) = 9.268; ε = 0.684; p < 0.001) were obtained. The pairwise group comparisons (see also figure 1) for the correct condition revealed a significantly better performance for h-L1 than for h-L2 (t(18.044) = 3.335; p = 0.011). No differences were observed for the semantically violated sentences. In the syntactic violation condition, d-L2 performed both worse than h-L1 (t(9.856) = 3.909; p = 0.003) and h-L2 (t(12.106) = 2.820; p = 0.046).

**Table 1:** Mean percentages and standard errors of correct responses.

groups sentences	h-L1 (n = 12)	h-L2 (n = 12)	d-L2 (n = 8)
correct	94.8% (0.9%)	88.7% (1.6%)	92.0% (1.2%)
semantic violation	97.6% (0.8%)	97.5% (0.8%)	94.2% (1.2%)
syntactic violation	95.5% (1.9%)	91.4% (2.6%)	77.3% (4.2%)

Represented are the mean percentages and standard errors of correct responses of the participants of the groups h-L1, h-L2, and d-L2 for the correct, semantically violated, and syntactically violated sentences.

### EEG Data

In the following sections the results of the ANOVAs for each of the three groups of participants, hearing native speakers of German (h-L1), hearing L2 learners of German (h-L2), and deaf L2 learners of German (d-L2) are presented. They are followed by between-group comparisons. Results for the semantic condition are always reported first, followed by results for the syntactic condition. The ANOVA model always comprised the within group factors Condition (CO), Hemisphere (HE), and Cluster (CL). For the between-groups ANOVAs the factor Group (GR) was added.

### Results Hearing German Native Speakers (h-L1)

#### Semantic Condition

**Figure 2:**
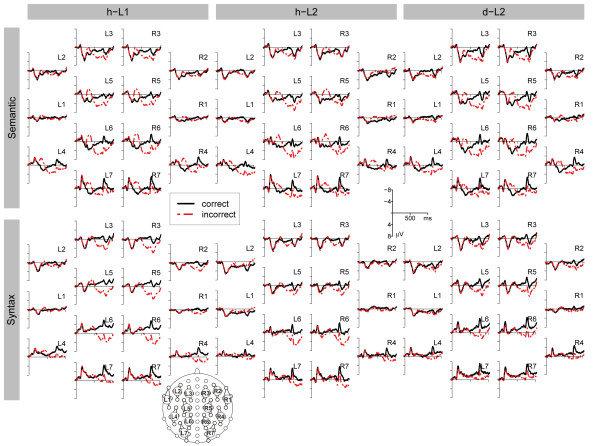
**Overview of the ERP results for all clusters**. Mean ERPs in the semantic (first row) and syntactic (second row) condition for h-L1 (first column), h-L2 (second column), and d-L2 (third column) for all clusters. The dotted line denotes the ERP of the incorrect condition, the solid line of the correct condition.

**Figure 3:**
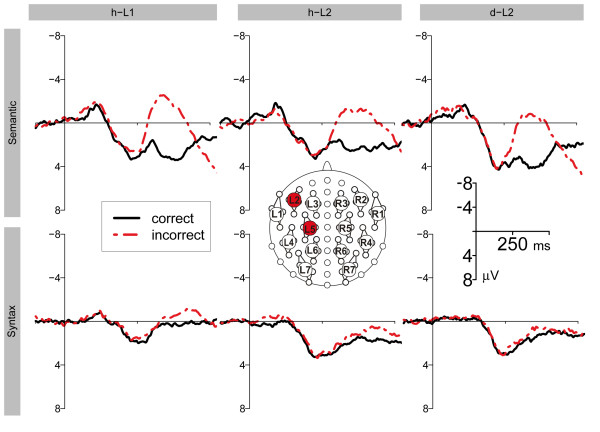
**Overview of the ERP results for selected clusters**. Mean ERPs in the semantic (first row) and syntactic (second row) condition for h-L1 (first column), h-L2 (second column), and d-L2 (third column) for chosen clusters (L5 for the semantic and L2 for the syntactic condition) that clearly showed an effect. The dotted line denotes the ERP of the incorrect condition, the solid line of the correct condition.

**Figure 4:**
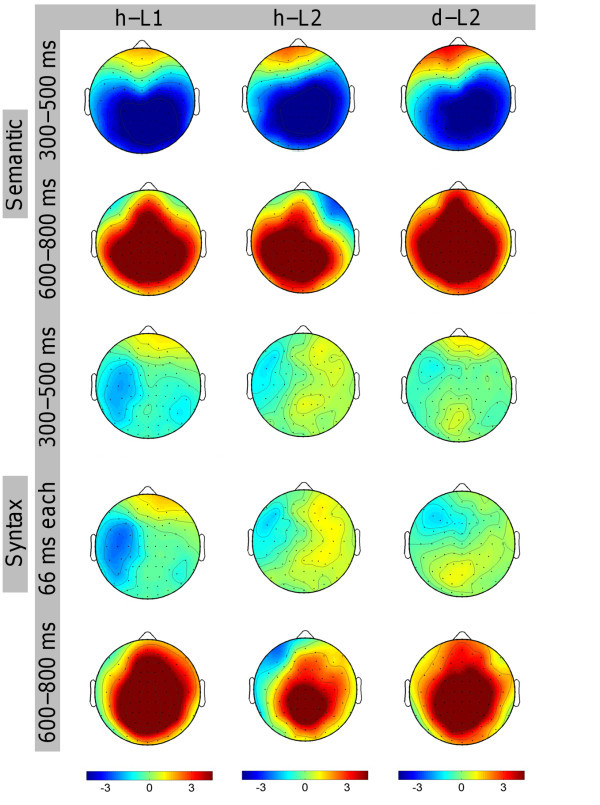
**Overview of the topographic distributions of the ERPs**. Topographies of the N400 (first row), semantic positivity (second row), syntactic negativity (third and fourth row), and P600 (fifth row) for h-L1 (first column), h-L2 (second column), and d-L2 (third column). Blue denotes negative values and red denotes positive values in μV. The annotation "66 ms each" denotes 300-366 ms for d-L2 and 366-433 ms for h-L1 and h-L2.

An ANOVA with mean amplitudes of the time epoch 300-500 ms as dependent variable, revealed a significant main effect of CO (F(1, 11) = 46.717; p < 0.001) and an interaction of CO and CL (F(2.3, 24.9) = 17.399; ε = 0.377; p < 0.001). The difference potential between ERPs elicited by semantic violations compared to the ERPs elicited by correct sentences were negative (p < 0.05) for all clusters except for the non significant clusters L2 and L3 (see figure 2, figure 3, and figure 4).

ERPs to semantic violations compared to semantically fitting words were significantly more positive going for the time epoch 600-800 ms (F(1, 11) = 22.307; p < 0.001). The interaction of CO and CL (F(1.8, 19.9) = 16.294; ε = 0.302; p < 0.001) was significant as well, indicating a symmetric centroparietal distribution of this effect. The difference between incorrect and correct words was positive for all clusters (p < 0.05) except L1 and R2 (see figure 2, figure 3, and figure 4).

#### Syntactic Condition

The ANOVA for mean amplitudes of the time epoch 300-500 ms revealed an interaction of CO and HE (Hemisphere) (F(1, 11) = 11.162; p = 0.007) indicating a left lateralized effect. The difference between the incorrect and the correct condition was significant for clusters L1, L2, L4, L5, and R4 (p < 0.05) (see figure 2, figure 3, and figure 4).

In addition, we divided the 300-500 ms time epoch into three sub epochs. We did not find any significant effect for the 300-366 ms epoch. For the 366-433 ms epoch we observed interactions of CO and HE (F(1, 11) = 23.788; p < 0.001) and CO, HE, and CL (F(2.1, 23.6) = 4.899; ε = 0.358; p = 0.015). Finally, a main effect of CO (F(1, 11) = 6.605; p = 0.026) and an interaction of CO and CL (F(2.2, 23.8) = 5.191; ε = 0.360; p = 0.012) were obtained for the 433-500 ms epoch (see figure 2, figure 3, and figure 4).

The ANOVA for mean ERP amplitudes of the time epoch 600-800 ms resulted in a main effect of CO (F(1, 11) = 38.299; p < 0.001) and interactions of CO and HE (F(1, 11) = 8.541; p = 0.014), CO and CL (F(2.3, 24.8) = 39.924; ε = 0.376; p < 0.001), and CO, HE, and CL (F(1.7, 18.6) = 3.939; ε = 0.282; p = 0.434). The P600 effect was significant for clusters L2-L7 and R1-R7 (p < 0.05) (see figure 2, figure 3, and figure 4).

### Summary Hearing German Native Speakers

In summary, h-L1 displayed an N400 effect to semantic violations which had a symmetric centroparietal scalp distribution. In addition, semantic violations elicited a symmetrically distributed posterior positivity following the N400.

The syntactic violation elicited a left lateralized negativity. This negativity was followed by a medially distributed P600.

### Results Hearing L2 learners of German (h-L2)

#### Semantic Condition

For the time epoch 300-500 ms, the ANOVA revealed a significant main effect of CO: ERPs to incorrect sentences were more negative than ERPs to correct sentences (F(1, 11) = 61.101; p < 0.001). Moreover, interactions of CO and CL (F(1.3, 13.9) = 16.086; ε = 0.210; p < 0.001), CO and HE (F(1, 11) = 6.771; p = 0.025), and CO, HE, and CL (F(2.0, 21.9) = 3.476; ε = 0.331; p = 0.049) were observed. The N400 effect was slightly lateralized to the right hemisphere. The negative difference was significant at clusters L4-L7, R1-R7; p < 0.05) (see figure 2, figure 3, and figure 4).

ERPs to incorrect sentences were significantly more positive than ERPs to correct sentences between 600-800 ms (F(1, 11) = 51.934; p < 0.001). The interactions of CO and CL (F(2.3, 25.6) = 29.653; ε = 0.388; p < 0.001), CO and HE (F(1, 11) = 9.913; p < 0.001), and CO, HE, and CL (F(2.0, 22.0) = 5.810; ε = 0.334; p = 0.009) were significant. The effect was stronger over the left than over the right hemisphere. Significantly positive ERP differences were revealed for all clusters except R1 and R2 (p < 0.05) (see figure 2, figure 3, and figure 4).

#### Syntactic Condition

Between 300-500 ms, an interaction of CO and HE (F(1, 11) = 6.320; p = 0.029) was observed. ERP differences between incorrect and correct sentences were negative over the left and positive over the right hemisphere. The interaction of CO, HE, and CL (F(2.1, 22.7) = 4.185; ε = 0.344; p = 0.027) was significant as well. The syntactic violation effect was significant at cluster L1 (p < 0.05) only (see figure 2, figure 3, and figure 4).

For the sub epoch 300-366 ms a main effect of CO (F(1, 11) = 5.222; p = 0.043) and an interaction of CO, and CL (F(2.5, 27.8) = 4.827; ε = 0.421; p = 0.011) were observed. For the remaining two sub epochs, interactions of CO and HE (366-433 ms: F(1, 11) = 7.095; p = 0.022; 433-500 ms: F(1, 11) = 5.875; p = 0.034), CO and CL (300-366 ms: F(2.5, 27.8) = 4.827; ε = 0.421; p = 0.011; 433-500 ms), and of CO, HE, and CL (366-433 ms: F(2.3, 24.8) = 4.020; ε = 0.376; p = 0.027; 433-500 ms: F(2.2, 24.0) = 4.210; ε = 0.363; p = 0.024) were significant (see figure 2, figure 3, and figure 4).

For ERPs between 600-800 ms, the main effect of CO (F(1, 11) = 23.533; p < 0.001) was significant, as were the interactions of CO and HE (F(1, 11) = 12.588; p = 0.005), CO and CL (F(1.8, 20.3) = 14.976; ε = 0.308; p < 0.001), and CO, HE and CL (F(2.8, 31.1) = 11.409; ε = 0.472; p < 0.001). This ERP effect had a right lateralized scalp topography. The violation effect was negative at cluster L1 and positive at clusters L5-L7 and R1-R7; p < 0.05) (see figure 2, figure 3, and figure 4).

### Summary Hearing L2 learners of German

The group of h-L2 showed an N400 following semantic violations which was stronger over the right than over the left hemisphere. The N400 was followed by a left lateralized positivity.

Syntactic violations elicited a left anterior negativity which was followed by a right lateralized P600 effect.

### Results Native Signers (d-L2)

#### Semantic Condition

For the time window 300-500 ms, the ANOVA revealed interactions of CO and CL (F(2.1, 14.7) = 6.883; ε = 0.351; p = 0.007), CO and HE (F(1, 7) = 9.011; p = 0.020), and CO, HE, and CL (F(1.7, 11.7) = 5.416; ε = 0.278; p = 0.026). This indicates a right lateralization of the N400 effect. Significant negative ERP differences were observed for the clusters L6, L7 and R1, R4, R5, R6, R7; p < 0.05) (see figure 2, figure 3, and figure 4).

For the time epoch 600-800 ms, ERPs to incorrect sentences were significantly more positive than ERPs to correct sentences (F(1, 7) = 11.999; p = 0.010). The interaction of CO and CL (F(1.9, 13.3) = 5.918; ε = 0.316; p = 0.016) was significant, as well. Positive ERP differences between incorrect and correct sentences were significant for clusters L3-L7 and R3-R7; p < 0.05) (see figure 2, figure 3, and figure 4).

#### Syntactic Condition

While the ANOVA for the time epoch 300-500 ms failed to show any CO effect, a significant interaction of CO and HE (F(1, 7) = 6.487; p = 0.038) was found for the sub epoch of 300-366 ms, The violation effect was significant for clusters L1 and L2 (p < 0.05). No further significant effects were obtained for the two following sub epochs (see figure 2, figure 3, and figure 4).

For the time epoch 600-800 ms, a main effect of CO (F(1, 7) = 8.865; p = 0.021) and an interaction of CO and CL (F(1.7, 12.2) = 4.889; ε = 0.291; p = 0.031) were significant. The ERP differences between incorrect and correct sentences were significantly positive for clusters L1, L3-L7, and R3-R7 (p < 0.05) (see figure 2, figure 3, and figure 4).

### Summary Native Signers

We observed an N400 effect for d-L2, which was more pronounced over the right than over the left hemisphere. The N400 effect was followed by a symmetrically distributed positivity. Syntactic violations elicited a left anterior negativity as well as a symmetrically distributed P600. A summary of the statistical results for each group is shown in table 2 for the semantic and table 3 and table 4 for the syntactic condition.

**Table 2:** ANOVAs for the semantic condition.

Semantics		Time epoch			
		
groups	effects	300-500 ms		600-800 ms	
		F	p	F	p
h-L1	CO	**46.717**	**< 0.001**	**22.307**	**<0.001**
	CO,HE	2.183	0.168	0.096	0.763
	CO,CL	**17.399**	**< 0.001**	**16.294**	**<0.001**
	CO,HE,CL	0.545	0.612	0.373	0.660
		F	p	F	p
h-L2	CO	**61.101**	**<0.001**	**51.934**	**<0.001**
	CO,HE	6.771	0.025	**9.913**	**<0.001**
	CO,CL	**16.086**	**<0.001**	**29.653**	**<0.001**
	CO,HE,CL	3.476	0.049	5.810	0.009
		F	p	F	p
d-L2	CO	4.943	0.062	11.999	0.010
	CO,HE	9.011	0.020	0.033	0.862
	CO,CL	**6.883**	**0.007**	5.918	0.016
	CO,HE,CL	5.416	0.026	0.296	0.741

CO: Condition, HE: Hemisphere, CL: Cluster; p < 0.05; **p < 0.01**; **p < 0.001**

The table contains the results of the Condition by Hemisphere by Cluster ANOVAs in the time epochs 300-500 ms and 600-800 ms for the groups h-L1, h-L2, and d-L2. Listed are all effects that included the factor Condition.

**Table 3:** ANOVAs for the syntactic condition.

Syntax		Time epoch			
		
groups	effects	300-500 ms		600-800 ms	
		F	p	F	p
h-L1	CO	4.414	0.059	**38.299**	**<0.001**
	CO,HE	**11.162**	**0.007**	8.541	0.014
	CO,CL	2.016	0.156	**32.924**	**<0.001**
	CO,HE,CL	2.473	0.106	3.939	0.043
		F	p	F	p
h-L2	CO	0.001	0.974	**23.533**	**<0.001**
	CO,HE	6.320	0.029	**12.588**	**0.005**
	CO,CL	0.534	0.602	**14.976**	**<0.001**
	CO,HE,CL	4.185	0.027	**11.409**	**<0.001**
		F	p	F	p
d-L2	CO	0.052	0.826	8.865	0.021
	CO,HE	1.076	0.334	0.241	0.638
	CO,CL	0.185	0.838	4.889	0.031
	CO,HE,CL	1.682	0.229	0.681	0.539

CO: Condition, HE: Hemisphere, CL: Cluster; p < 0.05; **p < 0.01**; **p < 0.001**

The table contains the results of the Condition by Hemisphere by Cluster ANOVAs in the time epochs 300-500 ms and 600-800 ms for the groups h-L1, h-L2, and d-L2. Listed are all effects that included the factor Condition.

**Table 4:** ANOVAs for three time epochs in the syntactic condition.

LAN		Time epoch					
		
groups	effects	300-366 ms		366-433 ms		433-500 ms	
		F	p	F	p	F	p
h-L1	CO	1.642	0.226	2.303	0.157	6.605	0.026
	CO,HE	3.056	0.108	**23.788**	**<0.001**	1.755	0.212
	CO,CL	0.484	0.587	1.130	0.343	5.191	0.012
	CO,HE,CL	1.434	0.260	4.899	0.015	1.133	0.344
		F	p	F	p	F	p
h-L2	CO	5.222	0.043	0.112	0.744	4.839	0.050
	CO,HE	2.135	0.172	7.095	0.022	5.875	0.034
	CO,CL	4.827	0.011	0.489	0.620	0.663	0.510
	CO,HE,CL	2.318	0.116	4.020	0.027	4.210	0.024
		F	p	F	p	F	p
d-L2	CO	0.026	0.877	0.330	0.584	<0.001	0.999
	CO,HE	6.487	0.038	0.006	0.939	0.146	0.714
	CO,CL	1.403	0.279	0.333	0.716	0.472	0.637
	CO,HE,CL	3.551	0.065	1.642	0.230	0.469	0.575

CO: Condition, HE: Hemisphere, CL: Cluster; p < 0.05**; p < 0.01**; **p < 0.001**

The table contains the results of the Condition by Hemisphere by Cluster ANOVAs in the time epochs 300-366 ms, 366-433 ms, and 433-500 ms for the groups h-L1, h-L2, and d-L2. Listed are all effects that included the factor Condition.

### Group Comparisons

#### Semantic Condition

The ANOVA with the between participant factor Group (GR) and the within participant factors Condition (CO), Hemisphere (HE), and Cluster (CL) revealed no significant effects for the time epoch 300-500 ms neither for all three groups nor for any combination of two groups.

The comparison of all three groups for the time window of 600-800 ms yielded a significant interaction of GR, CO, and HE (F(2, 29) = 3.884; p = 0.032).

The separate ANOVAs for pairwise comparisons of two groups revealed an interaction of GR, CO, and HE (F(1, 22) = 8.136; p = 0.009) as well as an interaction of GR, CO, HE, and CL (F(2.3, 50.3) = 3.178; ε = 0.381; p = 0.044) for the comparison of h-L1 and h-L2. The violation effect was stronger for h-L2 than for h-L1 over the left hemisphere. All statistical results of the group comparisons for the semantic condition are shown in table 5. The topographies are shown in figure 4.

**Table 5:** Group comparisons for the semantic condition.

Group Comparisons Semantics		time epoch			
		
groups	effects	300-500 ms		600-800 ms	
		F	p	F	p
h-L1/h-L2	GR,CO	0.007	0.932	0.527	0.475
	GR,CO,HE	0.957	0.339	**8.136°^**	**0.009°^**
	GR,CO,CL	0.502	0.593	0.691	0.524
	GR,CO,HE,CL	1.847	0.161	3.178°^	0.044°^
		F	p	F	p
h-L1/d-L2	GR,CO	0.360	0.556	0.357	0.558
	GR,CO,HE	1.687	0.210	0.107	0.747
	GR,CO,CL	0.086	0.936	0.222	0.792
	GR,CO,HE,CL	2.485	0.088	0.519	0.619
		F	p	F	p
h-L2/d-L2	GR,CO	0.449	0.511	1.621	0.219
	GR,CO,HE	0.100	0.756	3.100	0.095
	GR,CO,CL	0.103	0.894	1.242	0.302
	GR,CO,HE,CL	0.474	0.640	1.232	0.304

GR: Group, CO: Condition, HE: Hemisphere, CL: Cluster; p < 0.05; **p < 0.01**; **p < 0.001**

°: confirmed with z-score standardized values; ^: confirmed with normalized values

The results of the Group by Condition by Hemisphere by Cluster ANOVAs that included the factors Group and Condition are listed for the time epochs 300-500 ms and 600-800 ms for all group comparisons. Effects that were confirmed after z-standardization/vector normalization are indicated with a °/^ respectively.

#### Syntactic Condition

For time window 300-500 ms, the ANOVAs with all three groups or any pairwise comparison did not reveal any significant GR and CO effect.

Further dividing this time epoch into three sub epochs showed that h-L1 differed from h-L2 for 300-366 ms (GR and CO: F(1, 22) = 6.493; p = 0.018, GR, CO, and CL: F(2.4, 52.9) = 3.381; ε = 0.401; p = 0.034) and from d-L2 for 366-433 ms (GR, CO, and HE: F(1, 18) = 9.504; p = 0.006). The significant differences between h-L1 and h-L2 for 300-366 ms and between h-L1 and d-L2 for 366-433 ms were expected since in both cases one of the groups showed a LAN and the other did not show a significant LAN effect within the interval. Thus, they cannot be interpreted unequivocally.

As reported above, a negativity to syntactic violations was significant for sub epoch 366-433 ms in h-L1, for sub epoch 366-433 ms in h-L2 and for sub epoch 300-366 ms in d-L2. For a second topographic analysis we, therefore, compared these different time epochs between groups: The ANOVA comparing the three groups revealed a stronger left lateralized negativity for h-L1 compared to d-L2 (GR, CO, HE: F(1, 18) = 5.671; p = 0.028). No other comparisons reached significance level.

In the ANOVA with three groups no significant effects were observed for the time epoch 600-800 ms. The ANOVA with groups h-L1 and h-L2 revealed an interaction of GR and CO (F(1, 22) = 6.913; p = 0.015) indicating a more pronounced P600 of h-L1 than h-L2. The comparison of h-L1 and d-L2 did not show any significant effect.

The ANOVA with groups h-L2 and d-L2 revealed an interaction of GR, CO, and HE (F(1, 18) = 4.870; p = 0.041) and GR, CO, HE, and CL (F(3.4, 60.8) = 3.460; ε = 0.563; p = 0.018). The P600 was right lateralized for h-L2 but symmetrically distributed for d-L2. A post-hoc analysis^1 ^suggested that the topographical differences of h-L2 and both other groups are mainly due to a sustaining significant negativity at frontal clusters for h-L2 only. For an overview of all group comparison ANOVAs in the syntactic condition see table 6 and table 7. The topographies are shown in figure 4.

**Table 6:** Group comparisons for the syntactic condition.

Group Comparisons Syntax		time epoch			
		
groups	effects	300-500 ms		600-800 ms	
		F	p	F	p
h-L1/h-L2	GR,CO	2.057	0.166	6.913°^	0.015°^
	GR,CO,HE	0.351	0.560	0.119	0733
	GR,CO,CL	1.139	0.331	1.639	0.199
	GR,CO,HE,CL	0.301	0.761	0.913	0.417
		F	p	F	p
h-L1/d-L2	GR,CO	0.597	0.450	0.545	0.470
	GR,CO,HE	2.775	0.113	3.222	0.089
	GR,CO,CL	0.817	0.460	1.107	0.342
	GR,CO,HE,CL	0.360	0.701	1.044	0.364
		F	p	F	p
h-L2/d-L2	GR,CO	0.056	0.815	1.358	0.259
	GR,CO,HE	2.350	0.143	4.870°^	0.041°^
	GR,CO,CL	0.048	0.964	0.259	0.788
	GR,CO,HE,CL	0.413	0.680	3.460°^	0.018°^

GR: Group, CO: Condition, HE: Hemisphere, CL: Cluster; p < 0.05; **p < 0.01**; **p < 0.001**

°: confirmed with z-score standardized values; ^: confirmed with normalized values

The results of the Group by Condition by Hemisphere by Cluster ANOVAs that included the factors Group and Condition are listed for the time epochs 300-500 ms and 600-800 ms for all group comparisons. Effects that were confirmed after z-standardization/vector normalization are indicated with a °/^ respectively.

**Table 7:** Group comparisons for three time epochs in the syntactic condition.

Group Comparisons LAN		time epoch							
		
groups	effects	300-366 ms		366-433 ms		433-500 ms		300-366 ms vs 366-433 ms	
		F	p	F	p	F	p	F	p
h-L1/h-L2	GR,CO	6.493°	0.018°	1.732	0.202	0.049	0.826	-	-
	GR,CO,HE	0.639	0.433	0.021	0.885	0.607	0.444	-	-
	GR,CO,CL	3.381^	0.034^	0.752	0.487	1.174	0.319	-	-
	GR,CO,HE,CL	0.384	0.700	0.168	0.880	0.526	0.622	-	-
		F	p	F	p	F	p	F	p
h-L1/d-L2	GR,CO	0.330	0.573	0.107	0.748	1.215	0.285	0.740	0.401
	GR,CO,HE	0.743	0.400	**9.504**	**0.006**	0.581	0.456	5.671	0.028
	GR,CO,CL	0.990	0.372	0.690	0.531	0.627	0.550	1.934	0.155
	GR,CO,HE,CL	0.725	0.487	1.370	0.266	0.204	0.825	0.619	0.551
		F	p	F	p	F	p	F	p
h-L2/d-L2	GR,CO	2.162	0.159	0.499	0.489	0.921	0.350	0.113	0.741
	GR,CO,HE	0.083	0.777	3.732	0.069	2.449	0.135	1.762	0.201
	GR,CO,CL	1.201	0.316	0.267	0.800	0.343	0.716	0.571	0.591
	GR,CO,HE,CL	0.535	0.614	0.782	0.488	0.566	0.573	0.227	0.833

GR: Group, CO: Condition, HE: Hemisphere, CL: Cluster; p < 0.05; **p < 0.01**; **p < 0.001**

°: confirmed with z-score standardized values; ^: confirmed with normalized values

The results of the Group by Condition by Hemisphere by Cluster ANOVAs that included the factors Group and Condition are listed for the time epochs 300-500 ms and 600-800 ms for all group comparisons. Effects that were confirmed after z-standardization/vector normalization are indicated with a °/^ respectively.

### Comparisons of group topographies

In order to verify the topographic group differences for semantic and syntactic violation effects, the difference waves of ERPs to correct words subtracted from ERPs to violations were both normalized (vector normalization) and z-score standardized.

### Summary group comparisons

Semantic violations elicited a N400 followed by a positivity in all groups. The N400 topographies did not differ between groups. The positivity following semantic violations at 600-800 ms showed a more left lateralized distribution in group h-L2 compared to the symmetrical distribution in h-L1, none of the other comparisons revealed significant differences.

The topography of the negativity following syntactic violations did not differ significantly between groups for the epoch 300-500 ms. By analyzing the sub epoch, i.e. the sub epoch with the most robust syntax effect in each group, we found a left lateralization in each group. However, in the h-L1 the effect extended more towards temporal areas. The following P600 effect was very similar across groups.

## Discussion

The present study compared the neural correlates of intra- and crossmodal L2 acquisition. Behavioural and electrophysiological data were recorded from deaf (d-L2) and hearing (h-L2) L2 learners of written German, as well as from a group of hearing German native speakers (h-L1). Participants had to read and evaluate German sentences which were either correct or comprised a semantic or a syntactic violation. The ERP results revealed an N400 effect followed by a positivity for semantic violations in all three groups of participants. Syntactic violations elicited a left lateralized negativity and a subsequent P600. Thus, our data provide evidence that neural systems mediating intra- and crossmodal L2 acquisition do not principally differ but rather are astonishingly similar.

For the behavioural task, four, out of twelve, d-L2 were not able to reach the inclusion criterion of 60% correct responses for each condition, whereas all participants from the other two groups reached this criterion and were, thus, included in the EEG analyses. To account for this, we first discuss the EEG data of the high performing participants. A section which covers the behavioural data follows.

In the semantic condition, all groups showed a significant N400 effect. Semantic processes, as reflected in the N400, have been found to be generally less vulnerable than syntactic processes [e.g. [2]]. Our findings are in accord with the single existing previous study on deaf native signers processing a written L2: Neville et al. [57] found that an N400 after semantically implausible words in English was similar in deaf native signers of ASL compared to hearing native speakers. Additionally, we found a late positivity following the N400 for all three groups. A positivity after semantic violations has been reported in previous studies [12,23,58]. In a recent review, Kuperberg [14] suggested that positivities after semantic violations seem to be particularly likely when the sentence must be reanalysed immediately following the semantic violation. In the present study, the use of an acceptability judgement task might have favoured such a processing strategy. Furthermore, the sentence medial position of the violation and the low complexity of the sentences possibly allowed for an immediate reanalysis, as well.

While processing syntactic aspects of German, h-L1 showed a left lateralized negative ERP effect over the anterior and temporal cortex. Both d-L2 and h-L2 displayed a significant negativity which was most pronounced over the left anterior electrode sites, as well. The negativity was followed by a posteriorly distributed P600 effect, which was present in all three groups of participants.

The comparison of the ERPs of deaf and hearing L2 learners allowed us to decide between the hypothesis that a competition between signed and spoken languages prevents a full acquisition of an oral language, on the one side, and the alternative hypothesis that the acquisition of any L1, be it signed or spoken, can build a necessary foundation for learning a subsequent language, on the other side. In favour of the competition hypothesis, there have been a number of reports showing that the pre-implantation resting level and crossmodal activation level of auditory brain areas were negatively correlated with the degree of speech comprehension achieved with a cochlear implant [5]. Interestingly, such negative correlations were observed for a change of scalp topography of visual evoked potentials elicited by visual motion stimuli, as well [59]. The present result, however, suggests that learning a signed instead of an oral language first, results in a comparable functional organization of the L2. This finding is inconsistent with the competition hypothesis. Thus, it might be speculated that crossmodal reorganizations of visual processing mechanisms might interfere with the functional recovery of *auditory *processing including speech. By contrast genuine language functions, such as semantics and syntax might use overlapping neural systems that are equally assessed by the languages irrespective of the modality through which they have been learned [46,47,53].

The present study provides strong evidence for this assumption: The group of d-L2 exhibited semantic and syntactic ERPs in their L2 that were comparable to the ERPs of hearing L2 learners of German, even though German and DGS mediate genuine language functions in a structurally different manner. For example, grammatical information in DGS is predominantly spatially encoded. Further, we expected to see considerably more developmental vulnerabilities for the syntactic aspects of language processing than for semantic aspects, as suggested by a number of previous studies [2,29-31,57]. The occurrence of a LAN in deaf L2 learners is particularly meaningful since this ERP effect is known to be highly vulnerable, even for L1 speakers [39], for whom various different topographies have been reported [21,23,26-28]. Despite the relatively small number of participants in the group of deaf signers compared to neurolinguistic studies on healthy individuals, we obtained a significant LAN effect in d-L2, underscoring that deaf native signers are capable of activating brain systems that are important for the processing of syntactic aspects of oral languages.

In this context, it has to be noted that, in contrast to many ERP studies on L2 acquisition [29,30,35,36], both of our L2 learner groups started to learn German quite early (on average at the age of seven years). We suppose that both groups of L2 learners were able to acquire the syntactic processes indicated by the LAN because their AoA was, though delayed, still relatively early. Thus both, the early AoA and the high level of competence reached (note that only the high performing d-L2 were included in the EEG experiment), may be related to the elaboration of a processing module allowing an automatic parsing of written sentences of the L2, although both factors may be partially mutually dependent. Indeed, syntax related negativities and, thus, automatic parsing processes have been shown in other studies on verb-agreement violations in an L2 that was learned to a high proficiency [34-36,60].

Thus, our findings are in accord with the conclusions of Neville et al. [57], who supposed that grammar acquisition must have occurred before adolescence. In a sample of deaf native signers who had learned English after their early teens, Neville et al. [57] found that ERPs after closed class words, i.e. words that convey grammatical information, deviated qualitatively form the ERPs of hearing native speakers of English. Particularly, the left anteriorly distributed N280 was absent in deaf native signers of ASL. Indeed, our participants started to learn their written L2 at a much earlier age (at age six or seven) than Neville et al.'s participants. Interestingly, however, those deaf participants that had mastered the essentials of English grammar displayed an N280 which, moreover, was left lateralized. Unfortunately, this study (as our study) does not yet allow for drawing conclusions why not all native signers achieve the same competence in their (oral) L2.

We expected h-L1 to make fewer judgement errors than both L2 groups and the performance of h-L2 and d-L2 to be comparable. Indeed, h-L1 made fewer judgement errors in the correct condition than h-L2. They moreover performed at a higher level than d-L2 in all three conditions. The d-L2 performed worse than h-L2 in the syntactic condition, as well. It has to be noted that four deaf participants had to be excluded from the data analyses due to not reaching an accuracy level of 60% correct responses. Thus, our data are not in total agreement with the results of related studies [54]. Among others, Mayberry and Lock reported that deaf native signers of ASL performed equally well in an English language test as hearing L2 learners [54]. This inconsistency may be resolved by considering that having acquired a native language on time does not guarantee high levels of L2 proficiency. Additionally, it requires engaging with and in the L2. It is important to note considerable differences with regard to the development of literacy of the deaf in the USA and in Germany: In Germany the ideal of articulation practice and lip reading drills have dominated the classroom situation for a long time, especially in the generation of our participants [61]. In 1993, Nett and Wudtke [62] and Wudtke [63,64], respectively, have reported that the percentage of deaf pupils that reached a reading level appropriate for their age was as low as 5%. This educational history of our participants might account for the overall lower performance of d-L2 compared to h-L2 and particularly for the relatively high performance-related drop-out rate in the group of deaf native signers. This observation is reinforced by Chamberlain and Mayberry [65], who found that English reading proficiency of deaf signers of ASL was predicted by competence in ASL and, crucially, reading frequency. This indicates that an educational approach emphasizing articulation and lip reading at the expense of sign language may have discouraged many deaf pupils from engaging fully and frequently with reading materials. It provides a possible explanation for the huge inter-individual differences in reading proficiency among deaf native signers. In any case, our conclusions are restricted to relatively high proficient L2 learners. A generalization to the whole population of deaf native signers is not possible.

Another major difference between deaf and hearing L2 learners of German has to be noted: the deaf participants learned German predominantly over the visual channel. They generally do not acquire expressive language to the same degree as hearing L2 learners. Thus, the effects of the impoverished L2 learning opportunities might become more obvious for more complex sentences [62], as Felser [37] proposed. It remains an open question for further research whether group differences between hearing and deaf groups and/or between native speakers and L2 learners would emerge with complex sentence constructions. Nonetheless, our data provide clear evidence that language systems necessary to process semantic and syntactic aspects of language can, in principle, be set up and shaped by a sign language such that they can later be assessed by oral (written) languages.

## Conclusion

From the present data we conclude that the neural correlates of L2 processing in individuals with a signed L1 highly resemble those of hearing L2 learners with a spoken L1. In a group of deaf native signers who acquired written German as their L2, we were able to observe all semantic and syntactic language-related ERP effects well known from native speakers of oral languages. Especially the early syntactic negativity, indicating syntactic language functions particularly vulnerable to experience, was observed even in deaf participants who acquired their L2 crossmodally. It follows that the access to language processing systems is not competitive between modalities. Rather, the elaboration of these functions by input of one modality might facilitate the access by another modality, similarly to a monolingual context where good oral speech capacities (especially phonological skills) [66] are crucial for the development of high reading abilities [67]. Overall, it seems justified to conclude that intra- and crossmodal L2 acquisition involve comparable neural systems.

## Methods

The research was carried out in compliance with the Helsinki Declaration. The ethics committee of the German Society for Psychology (DGPS) approved the study (reference number: BRBHF 07022008).

### Participants

Profoundly congenitally deaf adults who had learned German Sign Language as their L1 on schedule and written German as an L2 (d-L2), hearing adults, who had learned an oral L1 and German as their L2 (h-L2) and hearing native speakers of German (h-L1) were compared in the present study. Participants received a monetary compensation. Participants of all three groups gave their voluntary informed consent for participation in an EEG experiment. They were recruited via written ads on announcement boards, on the web sites of the University of Hamburg, and, in case of d-L2, on different web sites of deaf communities. Additionally, on the web sites, a video in DGS informed them about the research project, in which they were asked to participate.

Participants with less than 60% correct responses in any condition were excluded from further analyses. Twelve h-L1 (three males; mean age: 31 years, median: 28 years, range: 22-53 years), twelve h-L2 (three males; mean age: 27 years, median: 25.5 years, range: 20-35 years), and twelve d-L2 took part. Eight d-L2 were included in the EEG analysis (four males; mean age: 28 years, median: 26 years, range: 21-40 years). The remaining four d-L2 were not included in the ERP analysis since they had less than 60% correct responses in at least one of the experimental conditions. The h-L2 started to acquire German at a comparable age (mean: 7 years median: 6.5 years, range: 4-11 years) as d-L2 who started to learn German at school when they were 6 or 7 years old. All participants were right handed and had normal or corrected to normal vision. Ten h-L1 had A-level ("Allgemeine Hochschulreife"), one O-level ("Fachhochschulreife"), and one a university degree; ten h-L2 had A-level, one a university degree, and one did not report his education level; seven of d-L2 had A-level (six were included), four O-level (one was included), and one did not report his education level. The native languages of the h-L2 were Bulgarian, Russian, Czech, Farsi, Turkish, Bosnian, Albanian, Polish, and English.

### Material

German sentences with either a semantic violation (implausible object) or a syntactic violation (verb-agreement) were used. All sentences had the following structure: (1) article, noun (subject)/(2) verb (predicate)/(3) article, noun (direct object)/(4) preposition, [an optional article], noun (prepositional phrase) (see table 8).

**Table 8:** Sentence examples.

*Condition*	*Example sentence*
Correct	Der Mann *kocht *das Essen in der Küche. Engl.: The man *cooks *the meal in the kitchen.
Syntactic verb-agreement violation	*Der Mann *kochen *das Essen in der Küche. Engl.: *The man *cook *the meal in the kitchen.
Semantic violation	*Der Mann kocht das Bild in der Küche. Engl.: *The man cooks the picture in the kitchen.

Correct, syntactically violated, and semantically violated sentences are shown. The critical word at which the verb-agreement violation was embedded is written in italic letters and the critical word at which the implausible object was embedded is written in underlined letters.

There were three different conditions: (1) correct, (2) semantically incorrect, and (3) morphosyntactically incorrect. Syntactic violations consisted of a subject-verb number agreement violation similar as in Osterhout and Mobley [18], Münte et al. [23], and De Vincenzi et al. [22].

The critical word in an incorrect sentence had a counterpart in its companion correct sentence with the same word class at the same position. Sentences with a morphosyntactic violation and sentences containing a semantic violation were derived from the same correct sentence. The use of errors in a sentence medial position eliminates the possible confounding influences of the linear distance between subject and verb, of phrase structure boundaries, of possible working memory load and of decision and motor processes [68].

The so called cloze probability of the direct objects was assessed in a pilot study. Each of the sentences described above were presented up to the article ("Der Mann kocht das ...") to a group of 104 students. They were asked to complete the sentence in the most expected way. Only sentences with a cloze probability of higher than 50% were included in the final sample (mean: 82.39%, SD: 14.35%). Semantic violations were generated by permuting the direct objects across sentences.

Eighty different sentences were generated comprising two sets of 40 sentences each (see table 2). The participants saw the sentences either in version A or in version B. The sentences in both versions were divided into set 1 (sentences 1-40) and set 2 (sentences 41-80). Each sentence in each set was presented 4 times, twice correctly (once with the subject in singular and once with the subject in plural), once syntactically incorrectly and once semantically incorrectly. The grammatical number of the subject of the sentences differed between the sets: In set A1, correct sentences with a plural subject were syntactically violated and sentences with a singular subject were semantically violated. Instead, in A2, correct sentences with a singular subject were syntactically violated and those with a plural subject were semantically violated. The correct sentences in version B1 were the same as the correct sentences in version A1. Similarly, sentences in B2 were the same as sentences in A2. However, the violations in B1 were the same as in A2 and violations in B2 were the same as in A1 (see table 9).

**Table 9:** Assignment of sentences to participants and conditions (n = 40 in each cell).

	Version A				Version B			
Set	Set 1 (A1)		Set 2 (A2)		Set 1 (B1)		Set 2 (B2)	

Correct	singular	plural	singular	plural	singular	plural	singular	plural

Violation	semantic singular	syntactic plural	syntactic singular	semantic plural	syntactic singular	semantic plural	semantic singular	syntactic plural

Additionally, 80 filler sentences were intermixed, half of which were correct and half were semantically or syntactically incorrect. Violations were embedded in the incorrect filler sentences at varying positions. Each participant was presented with a total of 400 sentences.

### Procedure

The sentences were presented in random order with black letters against a grey background with a vertical visual angle of 1.53°. A fixation cross appeared in the middle of the screen for 600 ms, followed by the words, which were presented at a rate of 600 ms per word. After the last word, a grey screen appeared for 600 ms, followed by a happy and a sad smiley. The participants had to decide whether or not the just seen sentence had been correct. The button on the side with the happy smiley had to be pressed when the sentence was correct and the button on the side of the sad smiley had to be pressed when the sentence was incorrect. The left and right index fingers were used for responding. Half of the participants saw the happy smiley on the right and the sad smiley on the left side, while the other half of the participants saw them in the reverse order. To start the presentation of the next sentence, the participants had to press one of the buttons. Sentences were presented in five blocks with 80 sentences each. After each block, a short pause was given. The experiment lasted for about 60 minutes.

### EEG Recording

**Figure 5:**
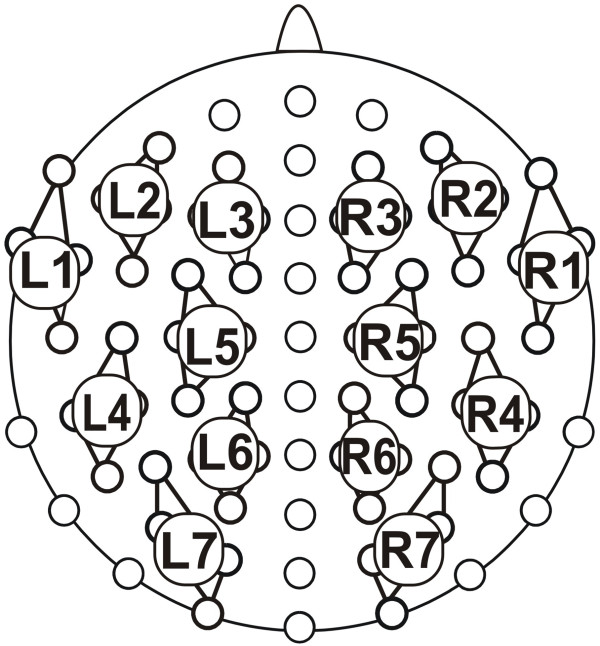
**Electrode montage and clustering**. The 14 clusters, 7 on each hemisphere, used for the statistical analyses are marked.

The electroencephalogram was recorded using 74 scalp electrodes mounted according to the international 10/10 system into an elastic cap (Easy Cap; FMS, Herrsching-Breitbrunn, Germany; see figure 5). All scalp electrodes were referenced to the right earlobe. An averaged right/left earlobe reference was calculated offline. For the statistical analyses of the ERP data, four adjacent electrodes were pooled, resulting in seven clusters for each hemisphere (see figure 5).

The vertical electrooculogram (VEOG) was measured with two electrodes placed under both eyes and recorded against the reference electrode. Horizontal eye movements were monitored using electrodes F9 and F10. Electrode impedance was kept below 5 kΩ.

The electrode signals were amplified using BrainAmp DC amplifiers (Brain Products GmbH, Gilching, Germany) and digitally stored using the BrainVision Recorder software (Brain Products GmbH, Gilching, Germany). The analog EEG signal was sampled at 5000 Hz, filtered online with a bandpass of 0.1 to 250 Hz and then downsampled online to 500 Hz to be stored on a disc. The signal was filtered offline with a high cut-off at 40 Hz, 12 dB/oct.

ERPs were averaged for the time periods between 100 ms prior to and 1500 ms after the critical words. Trials with erroneous responses were excluded. Moreover, trials with ocular artefacts were eliminated. An individually adjusted criterion between a peak-to-peak amplitude of 80 and 100 μV was used as the rejection threshold for ocular electrodes in each participant. A second artefact rejection iteration of alpha waves, muscular activity or drifts due to excessive sweating followed, which had an individually adjusted criterion up to 150 μV, as well. Remaining segments were baseline corrected with respect to a 100 ms period preceding the onset of the critical word. Separate averages were calculated for the four conditions: (1) semantically correct, (2) semantically incorrect, (3) syntactically correct, and (4) syntactically incorrect for each participant.

### Data Analysis

Mean amplitudes were calculated for the time periods 300-500 ms and 600-800 ms. The mean amplitudes were analysed by means of an ANOVA separately for each of the conditions semantics and syntax. In the syntax condition the time epoch 300-500 ms was further divided into the three sub epochs 300-366 ms, 366-433 ms, and 433-500 ms. Repeated measurement factors Condition (correct vs. incorrect), Hemisphere (left vs. right) and Cluster (one to seven) (henceforth: CO, HE, CL) and the between participant factor Group (h-L1, h-L2, d-L2) were included. Sums of Squares of Type II were calculated. To compensate for violations of the assumption of sphericity in multi-channel electroencephalographic data, the Greenhouse and Geisser correction was applied. Whenever a factor with more than two levels is involved, the Greenhouse and Geisser epsilon (ε) is reported.

Behavioural data were analyzed with an ANOVA comprising the between participant factor Group (h-L1, h-L2, and d-L2) and the within participant factor Condition (correct, semantically incorrect, and syntactically incorrect), which were predictor variables for the dependent variable percentage of correct responses. The degrees of freedom of the t-tests used to compare the three groups were corrected using the Welch algorithm. Due to the gap between the onset of the violation and the response of the participant, reaction times were not analysed. The open source statistical programming language "R" was used for statistical analyses.

## Authors' contributions

NS, MK, BHF, and BR designed the experiment. NS and MK run the ERP experiments. NS and BR analyzed the data. NS, MK, US, BHF, and BR wrote the paper. All authors read and approved the final manuscript.

## Endnotes

1 Running t-tests in 20 ms intervals for the incorrect condition minus the correct condition at clusters L1 and L2 were conducted between 600 and 1000 ms for h-L2. They showed significant negative effects (p < .05) for all intervals between 660 and 1000 ms at cluster L1. Negative effects in the interval 720-740 ms and all time epochs between 760 and 1000 ms reached significance at cluster L2. In h-L1 the single time epoch 620-640 ms revealed a significant positive effect at cluster L1. At cluster L2 intervals between 600 and 780 ms and 800-840 ms had significant positive effects. For d-L2 significant positive effects between 660 and 680 ms, 740-760 ms, and 780-800 ms at cluster L1; and 660-700 ms, 720-740 ms, and 880-900 ms at cluster L2 were revealed.

## References

[B1] Knudsen EI Sensitive periods in the development of the brain and behavior Journal of Cognitive Neuroscience 2004 16 1412 1425 10.1162/0898929042304796 15509387

[B2] Weber-Fox CM Neville HJ Maturational constraints on functional specialization for language processing: ERP and behavioral evidence in bilingual speakers Journal of Cognitive Neuroscience 1996 8 231 256 10.1162/jocn.1996.8.3.231 23968150

[B3] De Groot AMB Kroll JF Tutorials in bilingualism:Psycholinguistic perspectives 1997 Mahwah: NJ: Erlbaum

[B4] Wartenburger I Heekeren HR Abutalebi J Cappa SF Villringer A Perani D Early Setting of Grammatical Processing in the Bilingual Brain Neuron 2003 37 159 170 10.1016/S0896-6273(02)01150-9 12526781

[B5] Lee HJ Giraud AL Kang E Oh SH Kang H Kim CS Lee DS Cortical activity at rest predicts cochlear imlantation outcome Cerebral Cortex 2007 17 909 917 16731883 10.1093/cercor/bhl001

[B6] Perani D Paulesu E Galles NS Dupoux E Dehaene S Bettinardi V Cappa SF Fazio F Mehler J The bilingual brain. Proficiency and age of acquisition of the second language Brain 1998 121 1841 1852 10.1093/brain/121.10.1841 9798741

[B7] Kutas M Hillyard SA Reading senseless sentences: brain potentials reflect semantic incongruity Science 1980 207 203 205 10.1126/science.7350657 7350657

[B8] Chwilla DJ Brown CM Hagoort P The N400 as a function of the level of processing Psychophysiology 1995 32 274 285 10.1111/j.1469-8986.1995.tb02956.x 7784536

[B9] Holcomb PJ Neville HJ Nature speech processing: An analysis using event-related brain potentials Psychobiology 1991 19 286 300

[B10] Kutas M Hillyard SA Brain potentials during reading reflect word expectancy and semantic association Nature 1984 307 161 163 10.1038/307161a0 6690995

[B11] Hoeks JC Stowe LA Doedens G Seeing words in context: the interaction of lexical and sentence level information during reading Cognitive Brain Research 2004 19 59 73 10.1016/j.cogbrainres.2003.10.022 14972359

[B12] Kolk HH Chwilla DJ van Herten M Oor PJ Structure and limited capacity in verbal working memory: a study with event-related potentials Brain and Language 2003 85 1 36 10.1016/S0093-934X(02)00548-5 12681346

[B13] Kuperberg GR Sitnikova T Caplan D Holcomb PJ Electrophysiological distinctions in processing conceptual relationships within simple sentences Cognitive Brain Research 2003 17 117 129 10.1016/S0926-6410(03)00086-7 12763198

[B14] Kuperberg GR Sitnikova T Lakshmanan BM Neuroanatomical distinctions within the semantic system during sentence comprehension: evidence from functional magnetic resonance imaging Neuroimage 2008 40 367 388 10.1016/j.neuroimage.2007.10.009 18248739 PMC3141816

[B15] Coulson S King JW Kutas M Expect the unexpected: Event-related brain response to morphosyntactic violations Language and Cognitive Processes 1998 13 21 58 10.1080/016909698386582

[B16] Friederici AD Hahne A Mecklinger A Temporal structure of syntactic parsing: early and late event-related brain potential effects Journal of Experimental Psychology: Learning, Memory, and Cognition 1996 22 1219 1248 8805821 10.1037//0278-7393.22.5.1219

[B17] Neville H Nicol JL Barss A Forster KI Garrett MF Syntactically based sentence processing classes: evidence from event-related brain potentials Journal of Cognitive Neuroscience 1991 3 151 165 10.1162/jocn.1991.3.2.151 23972090

[B18] Osterhout L Mobley LA Event-related brain potentials elicited by failure to agree Journal of Memory and Language 1995 34 739 773 10.1006/jmla.1995.1033

[B19] Friederici AD Towards a neural basis of auditory sentence processing Trends in Cognitive Sciences 2002 6 78 84 10.1016/S1364-6613(00)01839-8 15866191

[B20] Felser C Clahsen H Munte TF Storage and integration in the processing of filler-gap dependencies: an ERP study of topicalization and wh-movement in German Brain and Language 2003 87 345 354 10.1016/S0093-934X(03)00135-4 14642537

[B21] Rodriguez-Fornells A Clahsen H Lleo C Zaake W Munte TF Event-related brain responses to morphological violations in Catalan Cognitive Brain Research 2001 11 47 58 10.1016/S0926-6410(00)00063-X 11240111

[B22] De Vincenzi M Job R Di Matteo R Angrilli A Penolazzi B Ciccarelli L Vespignani F Differences in the perception and time course of syntactic and semantic violations Brain and Language 2003 85 280 296 10.1016/S0093-934X(03)00055-5 12735945

[B23] Münte TF Matzke M Johannes S Brain activity associated with syntactic incongruencies in words and pseudo-words Journal of Cognitive Neuroscience 1997 9 318 329 10.1162/jocn.1997.9.3.318 23965010

[B24] Hagoort P Brown C Groothusen J The syntactic positive shift (SPS) as an ERP measure of syntactic processing Language and Cognitive Processes 1993 8 439 483 10.1080/01690969308407585

[B25] Osterhout L Holcomb PJ Swinney DA Brain potentials elicited by garden-path sentences: evidence of the application of verb information during parsing Journal of Experimental Psychology Learning Memory and Cognition 1994 20 786 803 10.1037//0278-7393.20.4.786 8064247

[B26] Kaan E Investigaing the effects of distance and number interference in processing subject-verb dependencies: an ERP study Journal of Psycholinguistic Research 2002 31 165 193 10.1023/A:1014978917769 12022794

[B27] Gross M Say T Kleingers M Clahsen H Munte TF Human brain potentials to violations in morphologically complex Italian words Neuroscience Letters 1998 241 83 86 10.1016/S0304-3940(97)00971-3 9507926

[B28] Weyerts H Penke M Munte TF Heinze HJ Clahsen H Word order in sentence processing: an experimental study of verb placement in German Journal of Psycholinguistic Research 2002 31 211 268 10.1023/A:1015588012457 12092710

[B29] Hahne A Friederici AD Processing a second language: Late learners' comprehension mechanisms as revealed by event-related brain potentials Bilingualism: Language and Cognition 2001 4

[B30] Chen L Shu H Liu Y Zhao J Li P ERP signatures of subject-verb agreement in L2 learning Bilingualism: Language and Cognition 2007 10 161 174 10.1017/S136672890700291X

[B31] Ardila A Language representation and working memory with bilinguals Journal of Communication Disorders 2003 36 233 240 10.1016/S0021-9924(03)00022-4 12742670

[B32] Hasegawa M Carpenter PA Just MA An fMRI study of bilingual sentence comprehension and workload Neuroimage 2002 15 647 660 10.1006/nimg.2001.1001 11848708

[B33] Ullman MA Sanz C. Georgetown A cognitive neuroscience perspective on second language acquisition: The declarative/procedural model Mind and Context in Adult Second Language Acquisition: Methods, Theory, and Practice 2005 University Press 141 178

[B34] Osterhout L McLaughlin J Kim A Greenwald R Inoue K Carreiras MaCCHE, Jr Sentences in the brain: event-related potentials as real-time reflections of sentence comprehension and language learning The online study of sentence comprehension : eyetracking, ERP, and beyond 2004 New York: Psychology Press 271 308

[B35] Tokowicz N MacWhinney B Implicit and explicit measures of sensitivity to violations in second language grammar: An event-related potential investigation Studies in Second Language Acquisition 2005 27 173 204

[B36] Hahne A Mueller JL Clahsen H Morphological processing in a second language: behavioral and event-related brain potential evidence for storage and decomposition Journal of Cognitive Neuroscience 2006 18 121 134 10.1162/089892906775250067 16417688

[B37] Clahsen H Felser C How native-like is non-native language processing? Trends in Cognitive Sciences 2006 10 564 570 10.1016/j.tics.2006.10.002 17071131

[B38] Rodriguez-Fornells A De Diego Balaguer R Münte TF Gullberg M, Indefrey P Executive control in bilingual language processing The cognitive neuroscience of second language acquisition 2006 Oxford: Blackwell 133 190

[B39] Osterhout L McLaughlin J Pitkänen I Frenck-Mestre C Molinaro N Novice learners, longitudinal designs, and event-related potentials: A means for exploring the neurocognition of second-language processing Language Learning 2006

[B40] De Diego Balaguer R Rodriguez-Fornells A Contributions to the functional neuroanatomy of morphosyntactic processing in L2 Language Learning 2010 60 231 259 10.1111/j.1467-9922.2009.00557.x

[B41] Clayton V Ceil L Linguistics of American sign language: an introduction 2000 3 Washington, DC: Gallaudet University Press

[B42] Bavelier D Newport EL Supalla T Signed or spoken, children need natural languages Cerebrum 2003 5 19 32

[B43] Emmorey K Reilly JS Language, gesture and space 1995 Hillsdale: Erlbaum

[B44] Goldin-Meadow S Mayberry RI How do profoundly deaf children learn to read? Learning Disabilities Research & Practice 2001 16 222 229 10.1111/0938-8982.0002221611985

[B45] Newport EL Maier RP D. Slobin The Acquisition of American Sign Language The Cross-Linguistic Study of Language Acquisition 1985 Erlbaum, Hillsdale, NJ 881 938

[B46] MacSweeney M Calvert GA Campbell R McGuire PK David AS Williams SC Woll B Brammer MJ Speechreading circuits in people born deaf Neuropsychologia 2002 40 801 807 10.1016/S0028-3932(01)00180-4 11900730

[B47] MacSweeney M Campbell R Woll B Brammer MJ Giampietro V David AS Calvert GA McGuire PK Lexical and sentential processing in British Sign Language Human Brain Mapping 2006 27 63 76 10.1002/hbm.20167 15966001 PMC6871280

[B48] Corina D The processing of sign language: Evidence from aphasia Handbook of neurolinguistics 1998 San Diego: Academic Press 313 329

[B49] Hickok G Bellugi U Klima ES What's right about the neural organization of sign language? A perspective on recent neuroimaging results Trends in Cognitive Sciences 1998 2 465 467 10.1016/S1364-6613(98)01263-7 21227293

[B50] Capek CM Grossi G Newman AJ McBurney SL Corina D Roeder B Neville HJ Brain systems mediating semantic and syntactic processing in deaf native signers: Biological invariance and modality specificity Proceedings of the National Academy of Sciences 2009 106 8784 8789 10.1073/pnas.0809609106 PMC2689005 19433795

[B51] Neville HJ Biological constraints on semantic processing: A comparison of spoken and signed languages Psychophysiology 1985 22 576

[B52] Mitchell TV Neville HJ Zani A Effects of age and experience on the development of neurocognitive systems The Cognitive Physiology of Mind 2002 Proverbio AM: Academic Press

[B53] Neville H Bavelier D Neural organization and plasticity of language Current Opinion in Neurobiology 1998 8 254 258 10.1016/S0959-4388(98)80148-7 9635210

[B54] Mayberry RI Lock E Age constraints on first versus second language acquisition: evidence for linguistic plasticity and epigenesis Brain and Language 2003 87 369 384 10.1016/S0093-934X(03)00137-8 14642540

[B55] Mayberry RI Lock E Kazmi H Linguistic ability and early language exposure Nature 2002 417 38 10.1038/417038a 11986658

[B56] Kutas M Neville HJ Holcomb PJ A preliminary comparison of the N400 response to semantic anomalies during reading, listening and signing Electroencephalography and Clinical Neurophysiology Supplement 1987 39 325 330 3477442

[B57] Neville HJ Mills DL Lawson DS Fractionating language: different neural subsystems with different sensitive periods Cerebral Cortex 1992 2 244 258 10.1093/cercor/2.3.244 1511223

[B58] van Herten M Kolk HH Chwilla DJ An ERP study of P600 effects elicited by semantic anomalies Cognitive Brain Research 2005 22 241 255 10.1016/j.cogbrainres.2004.09.002 15653297

[B59] Buckley KA Tobey EA Cross-modal plasticity and speech perception in pre- and postlingually deaf cochlear implant users Ear and Hearing 2011 32 2 15 20829699 10.1097/AUD.0b013e3181e8534c

[B60] Ojima S Nakata H Kakigi R An ERP study of second language learning after childhood: effects of proficiency Journal of Cognitive Neuroscience 2005 17 1212 1228 10.1162/0898929055002436 16197679

[B61] Poppendieker R Freies Schreiben und Gebärden. Voraussetzungen und Bedingungen des Erwerbs von Schreibkompetenz durch gehörlose Kinder Signum 1992

[B62] Nett O Wudtke H Schriftspracherwerb: Schreibentwicklungen gehörloser Kinder III Das Zeichen 1993 26 462 470

[B63] Wudtke H Schriftspracherwerb: Schreibentwicklungen gehörloser Kinder I Das Zeichen 1993 24 212 223

[B64] Wudtke H Schriftspracherwerb: Schreibentwicklungen gehörloser Kinder II Das Zeichen 1993 25 332 341

[B65] Chamberlaine C Mayberry RI American Sign Language syntactic and narrative comprehension in skilled and less skilled readers: Bilingual and bimodal evidence for the linguistic basis of reading Applied Psycholinguistics 2008 29 367 388

[B66] Bryant P Goswami U Phonological skills and learning to read (Essays in Developmental Pychology) 1990 Hove, East Sussex, UK: Psychology Press

[B67] Catts HW Kamhi AG The connections between language and reading disabbilities 2005 Mahwah, NJ.: Lawrence Erlbaum Associates

[B68] Osterhout L Nicol J On the distinctiveness, independence, and time course of the brain responses to syntactic and semantic anomalies Language and Cognitive Processes 1999 14 283 317 10.1080/016909699386310

